# Polyethylene Terephthalate Microplastic Exposure Induced Reproductive Toxicity Through Oxidative Stress and p38 Signaling Pathway Activation in Male Mice

**DOI:** 10.3390/toxics12110779

**Published:** 2024-10-25

**Authors:** Tianyang Li, Bohao Bian, Rihao Ji, Xiuwen Zhu, Xiaohui Wo, Qiankun Song, Zhigang Li, Feifei Wang, Yuqiao Jia

**Affiliations:** 1 State Key Laboratory of Environmental Criteria and Risk Assessment, Chinese Research Academy of Environmental Sciences, Beijing 100012, China; 15174954041@163.com (T.L.); zxw180607@163.com (X.Z.); lizg@craes.org.cn (Z.L.); 2 Hulunbuir Center for Disease Control and Prevention, Hulunbuir 021000, China; 13604749342@163.com (B.B.); 15548140059@163.com (X.W.); sqk860131@126.com (Q.S.); 3 School of Public Health, Baotou Medical College, Baotou 014000, China; jrh15548371105@163.com

**Keywords:** microplastics, polyethylene terephthalate, reproductive toxicity, oxidative stress, p38 signaling pathway

## Abstract

Polyethylene terephthalate (PET) is a type of polymer plastic that is often used to make plastic bags, bottles, and clothes. However, the waste of such plastic products is decomposed into microplastics (MPs), which are plastic fragments smaller than 5 mm, by various external forces such as wind, UV radiation, mechanical wear, and biodegradation. PET MPs have been widely detected in the environment and human tissue samples; however, the toxicity and mechanism of PET MPs in mammals are still unclear. In this study, we investigated the male reproductive toxicity of PET MPs and their underlying mechanism. A total of 80 male mice were orally exposed to 0.01, 0.1, and 1 mg/d of PET MPs (with a diameter of 1 μm) for 42 days. The results showed that 1 μm PET MPs induced different degrees of pathological damage to testicular tissues, decreased sperm quality, and increased the apoptosis of spermatogenic cells via oxidative stress and p38 signaling pathway activation. To further illustrate and verify the mechanistic pathway, oxidative stress was antagonized using N-acetylcysteine (NAC), and the activation of the p38 signaling pathway was blocked using SB203580. The results revealed that the male reproductive injury effects after exposure to PET MPs were significantly ameliorated. Specifically, the testicular tissue lesions were relieved, the sperm quality improved, and the apoptosis of spermatogenic cells decreased. These results demonstrated that PET MP exposure induced male reproductive toxicity through oxidative stress and the p38 signaling pathway. This study provides new insights into the reproductive toxicity of MPs in males, as well as valuable references for public health protection strategies.

## 1. Introduction

The term microplastics (MPs), defined by the United Nations Environment Programme (UNEP) as important emerging pollutants in the environment [1], was first proposed by Thompson et al. in 2004 [2] and has been extensively studied by many scholars [3,4,5,6]. Currently, plastic particles with a diameter of less than 5 mm are defined as MPs [7]. Many studies have shown that MPs are able to be widely distributed and spread in various environmental media [8,9,10]; MPs have been found in human foods and beverages, as well as in their packaging materials [11,12,13]. Relevant studies have revealed that different types of MPs are gradually being found in some tissues of the human body and might pose a potential risk to human health. The types of MPs detected in human blood included polyethylene terephthalate (PET), polyethylene (PE), and polystyrene (PS) [14]. A previous study found 12 types of MPs, such as PET and polypropylene (PP), in 13 human lung tissue samples [15]. In addition, semen samples from men in a polluted area of the Campania Region (Southern Italy) were collected, and PP, PE, PET, and PS were also detected, suggesting that MPs might be present in male gonads [16].

As MPs have been detected in human tissue samples, their toxic effects and mechanisms have become a hot topic. Some studies have focused on the damaging effects and mechanisms of inhaling PP MPs and PE MPs on the lung tissue [17], as well as those of oral microplastics (PE MPs) on the digestive system [18]. Moreover, MPs can enter the bloodstream [19] and can gradually be distributed throughout the body through blood circulation, causing damaging effects to health, including damage to the reproductive system [20]. Tissue oxidative stress is one of the important mechanisms related to the toxic effects of the body’s exposure to MPs. Relevant studies have shown that after oral exposure to PP MPs, antioxidant levels in the colon tissue of mice were reduced, and the oxidative stress response was further activated [21]. In the liver tissues of mice, PS MPs led to the disruption of oxidation/antioxidant balance by reducing antioxidant enzyme activity and ultimately aggravating oxidative stress [22]. Other studies have confirmed that the reproductive toxicity of PS MPs to male mice not only leads to a decrease in sperm quality, a disordered arrangement of spermatogenic cells, and testosterone level decline [23], but also induces testicular oxidative stress and causes testicular oxidative damage [24]. In addition, MP exposure can also activate the p38 signaling pathway. Moreover, the aforementioned study found that MP exposure induced p38 phosphorylation in testicular tissue, and the degree of phosphorylation increased with increasing concentrations of PS MP exposure [24].

PET, a type of polymer plastic, has the advantages of high strength and good heat resistance [25]; thus, it is commonly used in the manufacture of food packaging and textile materials [12,26,27]. PET MPs were one of the main microplastic components detected in tissue samples in vivo [14,15,16]. In the human gastrointestinal tract, PET MPs and their hydrolyzed products have been shown to aggravate the inflammatory response of macrophages, thus affecting intestinal homeostasis [28]. PET MPs pose a potential risk to human health. To date, studies on the reproductive toxicity of microplastics have mainly focused on PS MPs. However, the toxic effects of PET MPs on the mammalian reproductive system and its toxicity mechanism are unknown. Therefore, they need to be further explored.

In this study, the reproductive toxicity of PET MPs was explored in male mice based on sperm quality, testicular tissue pathological changes, spermatogenic cell apoptosis, and apoptosis factor expression. The mechanisms hypothesized in this study include oxidative stress and the p38 signaling pathway. Thus, we conducted an experiment in which the antioxidant NAC was used to antagonize oxidative damage, and SB203580 was used to block the p38 signaling pathway. In order to further explore the reproductive toxicity of PET MP exposure in male mice and its mechanism, our research focused on the following two questions: (1) Do PET MPs cause reproductive damage? (2) Is the reproductive toxicity induced by PET MPs related to oxidative stress and the p38 signaling pathway?

## 2. Materials and Methods

### 2.1. Microplastic Samples

PET MPs with a diameter of 1 μm (Shanghai Yang Li Electromechanical Technology Co., Shanghai, China) were configured to the experimental concentration with saline and were fully mixed using ultrasonic vibration treatment for 20 min. The standard particle size boundary used to distinguish MPs from NPs is 1 μm [7]. In addition, previous studies have shown that PS MPs with a size of 1 μm can enter the testicular tissue of mice, causing significant reproductive damage [23]. Therefore, a PET MP particle size of 1 μm was chosen as the subject of this study.

### 2.2. Animal and Ethics Statement

SPF male BALB/c mice (5 weeks old, weighing 18–22 g) were purchased from Peking University Health Science Center (Beijing, China) and were housed in the Animal Breeding Center of the School of Public Health, Baotou Medical College, where the temperature of the animal room was maintained at 22–26 °C and the relative humidity was 40–45%. During the feeding period, the mice were provided with sterile water and mouse food (Beijing Keao Xieli Feed Co., Ltd., Beijing, China) ad libitum. All mice care and experimental procedures followed the guidelines of ARRIVE (Animals in Research: In Vivo Experiments) and were approved by the Medical Ethics Review Committee of Baotou Medical College (Baotou Medical College, Animal Ethics 2021, Approval No. 024).

### 2.3. Experimental Design

A total of 80 mice were acclimatized in the animal room for one week and were then divided into 8 groups of 10 mice each. The units of measurement for humans and mice (mg/kg to mg/m^2^) were K_m_ (formula: mg/m^2^ = K_m_ × mg/kg), and K_m_ = kg/m^2^. As the average body weights are 60 kg (for humans) and 0.02 kg (for mice) and the body surface areas are 1.62 m^2^ (for humans) and 0.007 m^2^ (for mice), the K_m(human)_ and K_m(mouse)_ values are about 37 kg/m^2^ and 3 kg/m^2^, respectively [29]. Adults may consume 0.04–11.67 mg/kg of plastic particles per day [30,31]. Therefore, according to the formula “Mouse equivalent dose (mg/kg) = Human dose (mg/kg) × [K_m(human)_/K_m(mouse)_]”, the mouse equivalent dose is about 0.5–144 mg/kg [29], and the mouse dose (0.02 kg) is about 0.01–2.88 mg/day. Thus, the following three equivalent doses were selected: 0.01 mg/d, 0.1 mg/d, and 1 mg/d.

Moreover, for exploring the key role of oxidative stress and p38 signaling pathway activation in the reproductive toxicity of male mice as induced by PET MP exposure, the antioxidant NAC (MedChemExpress, Monmouth Junction, NJ, USA) was used to antagonize oxidative damage, and SB203580 (MedChemExpress, Monmouth Junction, NJ, USA) was used to specifically block the p38 signaling pathway. NAC is the precursor of glutathione (GSH), which has been proven to have a better antioxidant activity than GSH. As a common antioxidant, NAC can effectively cross various tissue barriers in the body, thus playing an important role in fighting oxidative stress damage [32,33]. SB203580 is an inhibitor of the cellular permeability of p38 MAPK, which has been reported to be able to effectively inhibit the activation of the p38 MAPK signaling pathway as a result of exposure to exogenous substances [34,35].

Hence, the experimental groups were as follows: (1) saline group (control group); (2) 0.01 mg/d PET MPs (low-dose group); (3) 0.1 mg/d PET MPs (medium-dose group); (4) 1 mg/d PET MPs (high-dose group); (5) 100 mg/kg/d NAC (NAC-treated group) [24]; (6) 1 mg/d PET MPs + NAC (NAC intervention group); (7) 5 mg/kg/d SB203580 (SB203580-treated group); and (8) 1 mg/d PET MPs + SB203580 (SB203580 intervention group).

### 2.4. Animal Experiments

The mice in group (1) served as the control group and were treated with sterile saline only, which was administered daily in an amount of 0.25 mL via the gavage method. Each mouse in groups (2), (3), and (4) was given 0.25 mL of PET MP solution at different concentrations daily using the gavage method, and each mouse in groups (6) and (8) was given 0.25 mL of PET MP solution at different concentrations daily using the gavage method. According to the body weight of each mouse, 100 mg/kg of NAC solution was given intraperitoneally to the mice in groups (5) and (6) every day as an antagonist of oxidative damage; the mice in groups (7) and (8) were intraperitoneally injected with SB203580 solution in an amount of 5 mg/kg once every four days. After 42 days of exposure [24], the reproductive system damage effects induced via PET MP exposure were detected.

### 2.5. Collection and Processing of Testicular Tissue Samples

After the animal experiments, euthanasia was performed via cervical dislocation. Then, the mice were immobilized, and their abdominal cavities were wiped with alcohol cotton balls. The abdominal cavities were cut open with ophthalmic scissors to locate the testes and epididymis on both sides. The testes and epididymis were placed in sterile saline, and the excess fatty tissue on the testes and epididymis was removed with scissors and forceps. The epididymis was isolated for the preparation of fresh sperm suspension. Finally, the testicles were fixed with 4% paraformaldehyde for indicator detection.

### 2.6. Testicular Histopathology

The testicular histopathological changes were detected using the hematoxylin and eosin (HE) staining method [23,24]. The fixed testicular tissue samples were rinsed and dehydrated using different concentration gradients of ethanol. Then, the samples were treated with alcohol benzene and xylene, and were then embedded in liquefied paraffin wax. Next, the wax blocks of embedded testicular tissues were sliced in a slicer, stained by adding hematoxylin staining solution, dehydrated again using ethanol in different concentration gradients, deparaffinized with xylene, and then blocked with neutral gum. Finally, the slices were observed, photographed, and analyzed under a light microscope.

### 2.7. Sperm Quality Assessment

The isolated epididymides were cut lengthwise with scissors in 9 mL phosphate buffer (PBS, pH = 7.2) and heated at 37 °C for 20 min to ensure that the sperm left the epididymal tubules. Firstly, 10 μL of homogeneous sperm suspension was placed on a cell counting plate and observed under a light microscope [24]. Then, 100 sperm were recorded, and the number of live sperm was quickly counted. Finally, the sperm suspension slide was fixed in methanol for 5 min and stained in 1% eosin solution for 1 h. After the excess eosin solution was washed off the surface and dried, the malformed sperm (fat head, double head, double tail, drongo, and/or unhooked and folded tail) were counted under a microscope among 1000 sperm [36]. In addition, the number of sperm was converted to the number of sperm per gram of epididymis. Sperm quality evaluation includes two indices—the rate of sperm viability and the rate of teratosperm. The rate of sperm viability was equal to the number of live sperm/100 × 100%. The rate of teratosperm was equal to the number of sperm malformations/1000 × 100%.

### 2.8. Spermatogenic Cell Apoptosis and the Expression of Apoptosis Factors

The apoptosis of spermatogenic cells was detected using the Terminal dUTP Nick End Labeling (TUNEL) staining method [37,38]. The testicular tissue sections were deparaffinized using xylene and were washed using a gradient of ethanol. Next, the tissues were treated with a Proteinase K working solution for 20 min, before being rinsed 3 times with PBS. Then, 50 μL of TUNEL reaction mix (50 μL of TdT enzyme + 450 μL of fluorescence reaction solution) was added to the tissues, and the slides were rinsed 3 times with PBS. After drying, 50 μL of converter POD was added to the tissues, and the reaction was carried out for 30 min in a dark humid environment. In the next step, the slides were rinsed 3 times with PBS, and 50–100 μL of DAB substrate was added to the tissues and reacted for 10 min. Finally, the tissues were rinsed 3 times with PBS and restained with hematoxylin. The morphology of apoptotic spermatogenic cells was observed under a light microscope. The number of apoptotic spermatogenic cells was counted using Image J. The rate of apoptotic cells was equal to the number of positive cells (apoptotic cells)/the number of total cells.

Aspartate-specific cysteine proteases (Caspases) are important effector factors in the molecular mechanism of apoptosis. Caspase molecules that are closely related to cell apoptosis include Caspase-3 and Caspase-9. The expression of apoptotic factors was detected using an enzyme-linked immunosorbent assay (ELISA) kit (Quanzhou Jiubang Biotechnology Co. Ltd., Quanzhou, China).

### 2.9. Oxidative Stress

The levels of MDA and reduced GSH in the testicular tissue were examined using a Reduced Glutathione (GSH) Colorimetric Assay Kit (Elabscience, Wuhan, China) and a Malondialdehyde (MDA) Colorimetric Assay Kit (Elabscience, Wuhan, China).

### 2.10. The Expression of Phosphorylated p38 Mitogen-Activated Protein (p-p38)

The p38 signaling pathway can mediate apoptosis by reducing the activity of anti-apoptotic genes [39], in which the relative expression of p-p38 can reflect the activation state of the p38 signaling pathway. The p-p38 protein quantification of testicular tissue was performed using the Western blot method. The p38 antibody (AF4001; 1:1000 dilution), p-p38 antibody (AF3455; 1:1000 dilution), and β-actin (AF7018; 1:1000 dilution) were purchased from Affinity (Santa Barbara, CA, USA). The samples were electrophoresed in an SDS-PAGE gel and were transferred to a PVDF membrane (Millipore, MA, USA). This was followed by closure using 5% skimmed milk powder. After washing, the membrane was incubated with the corresponding primary antibody at 4 °C overnight. After incubation, the membrane was rinsed 3 times, and the washed primary antibody was put into the secondary antibody working solution to undergo a reaction. After the membrane was rinsed 3 times, it was placed into an exposure cassette and was developed using the gel imaging system. Finally, Image J 1.8.0 software was used to analyze the grayscale.

### 2.11. Statistical Analysis

All data were expressed as mean ± SEM deviation, and statistical analyses were performed using the software GraphPad Prism 9.0.0. Multiple comparisons were performed using a one-way ANOVA analysis with the Tukey post hoc test; values of *p* < 0.05 and *p* < 0.01 were considered to be statistically significant.

## 3. Results

### 3.1. The Reproductive Damage Effects of PET MPs

**Figure 1:**
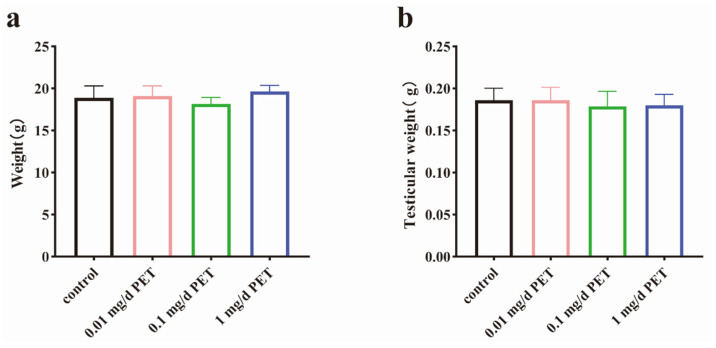
Body and testicular weight. (**a**) Body weight. (**b**) Testicular weight. *n* = 10 for all groups. Data are presented as the mean ± SEM.

As shown in Figure 1, there was no significant difference in weight change in terms of body weight (Figure 1a) and the testicular weight (Figure 1b) after six weeks of exposure to PET MPs.

**Figure 2:**
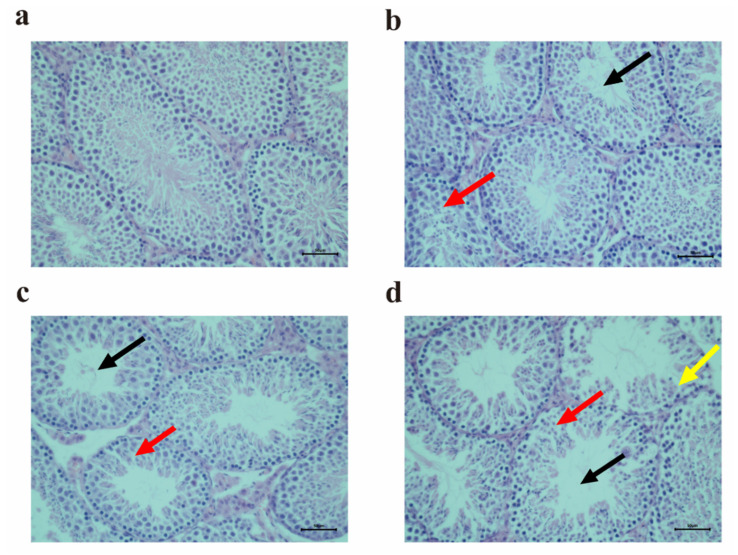
The pathological changes in testicular tissues determined via HE staining after PET MP exposure. (**a**) Control group; (**b**) 0.01 mg/d PET MP group; (**c**) 0.1 mg/d PET MP group; and (**d**) 1 mg/d PET MP group. The black arrow indicates that the spermatogenic tubule was empty. The red arrow indicates the disorder of the spermatogenic epithelium. The yellow arrow indicates the decrease in spermatogenic cells. Scale bar: 50 µm. *n* = 5 for all groups.

To assess the effects of PET MP exposure on testicular tissue in mice, the pathological changes in testicular tissue were observed, as shown in Figure 2. Compared with the control group, the morphological results showed the vacuolization of seminiferous tubules and the arrangement disturbances of the spermatogenic epithelium in all three dose groups (Figure 2b–d). Microscopically, the number of spermatogenic cells decreased in the high-dose group (Figure 2d). The findings indicated that exposure to PET MPs at different doses induced reproductive damage in the male mice, and the pathologic damage gradually worsened with the increasing dosage.

**Figure 3:**
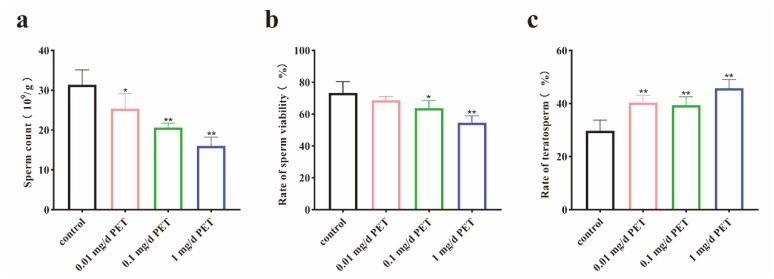
The changes in sperm quality after PET MP exposure. (**a**) The number of sperm; (**b**) the rate of sperm viability; and (**c**) the rate of teratosperm. *n* = 5 for all groups. Data are presented as the mean ± SEM. * indicates *p* < 0.05 (compared with the control group). ** indicates *p* < 0.01 (compared with the control group).

To further evaluate the reproductive capacity after PET MP exposure, the number of sperm, the rate of sperm survival, and the rate of sperm malformation were all determined. The results showed that the sperm counts in the different PET MP treatment groups significantly decreased in comparison to those in the control group, especially those in the medium- and high-dose groups (*p* < 0.01, Figure 3a). The sperm survival rate significantly decreased in the medium- and high-dose groups (*p* < 0.05, Figure 3b). In addition, the sperm deformity rate was significantly increased in all exposed groups (*p* < 0.01, Figure 3c).

### 3.2. The Apoptosis of Spermatogenic Cells and the Expression of Apoptosis Factors

**Figure 4:**
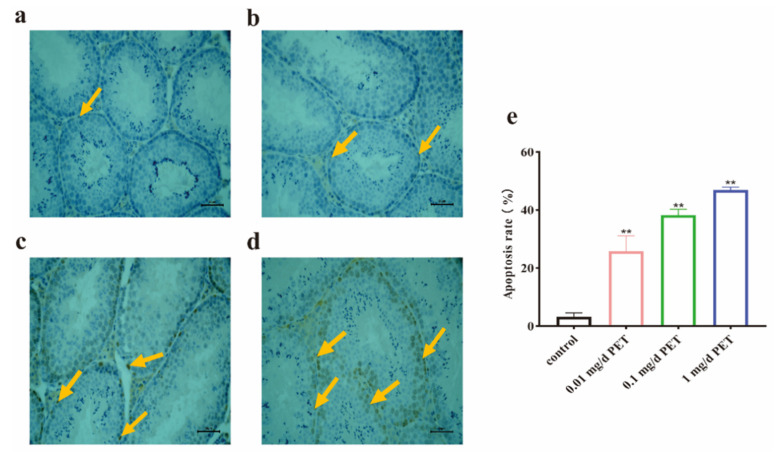
The effect of spermatogenic cell apoptosis via TUNEL staining after PET MP exposure. (**a**) Control group; (**b**) 0.01 mg/d PET MP group; (**c**) 0.1 mg/d PET MP group; (**d**) 1 mg/d PET MP group; and (**e**) the quantitative analysis of the apoptosis rate. The orange arrow indicates the apoptotic cells. Scale bar: 50 µm. *n* = 5 for all groups. Data are presented as the mean ± SEM. ** indicates *p* < 0.01 (compared with the control group).

Apoptosis is a basic phenomenon of life, and it maintains the dynamic balance of the number of cells in the body. However, an increase in spermatogenic cell apoptosis can cause damaging reproductive health effects. As shown in Figure 4, the effects of spermatogenic cell apoptosis induced by PET MPs were observed using TUNEL staining. The brown area of apoptotic spermatogenic cells in Figure 4b–d expanded compared with the blue area of normal cells in the control group (Figure 4a). Moreover, the apoptosis rate of spermatogenic cells was significantly increased in a dose-dependent manner (*p* < 0.01, Figure 4e).

**Figure 5:**
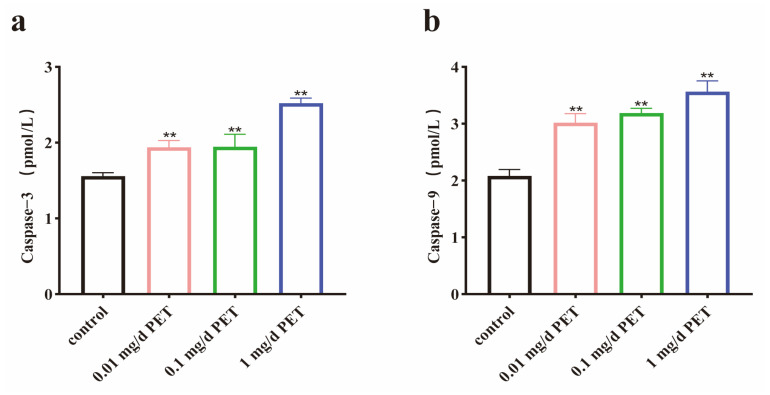
The expression levels of Caspase-3 and Caspase-9 after PET MP exposure. (**a**) The levels of Caspase-3; (**b**) the levels of Caspase-9. *n* = 5 for all groups. Data are presented as the mean ± SEM. ** indicates *p* < 0.01 (compared with the control group).

To further verify the effect of PET MP exposure on cell apoptosis, the levels of two apoptotic factors—Caspase-3 and Caspase-9—were examined. It was found that the expression levels of Caspase-3 (Figure 5a) and Caspase-9 (Figure 5b) were increased in the groups exposed to different doses of PET MPs (*p* < 0.01).

### 3.3. The Role of Oxidative Stress in Male Reproductive Toxicity Induced by PET MPs

**Figure 6:**
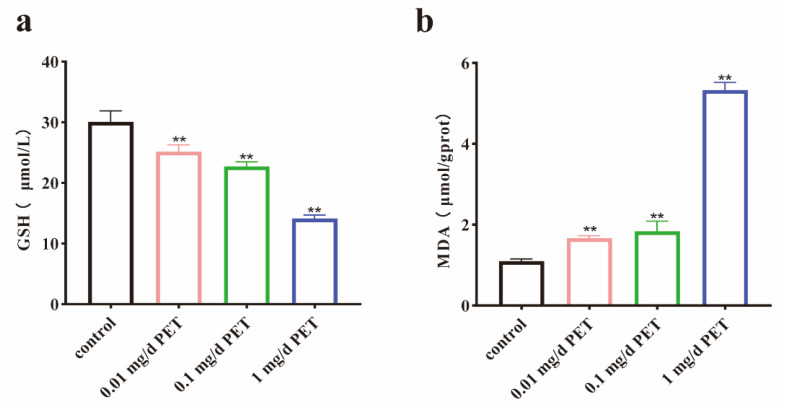
GSH and MDA levels in testicular tissue after PET MP exposure. (**a**) GSH levels; (**b**) MDA levels. *n* = 5 for all groups. Data are presented as the mean ± SEM. ** indicates *p* < 0.01 (compared with the control group).

As shown in Figure 6a,b, the GSH and MDA levels in the testicular tissues of the mice were detected after PET MP exposure. It was found that the GSH levels significantly decreased (*p* < 0.01) and the MDA levels significantly increased (*p* < 0.01), suggesting that the oxidative damage mechanism might play a role in the male reproductive health damage effects of PET MPs.

**Figure 7:**
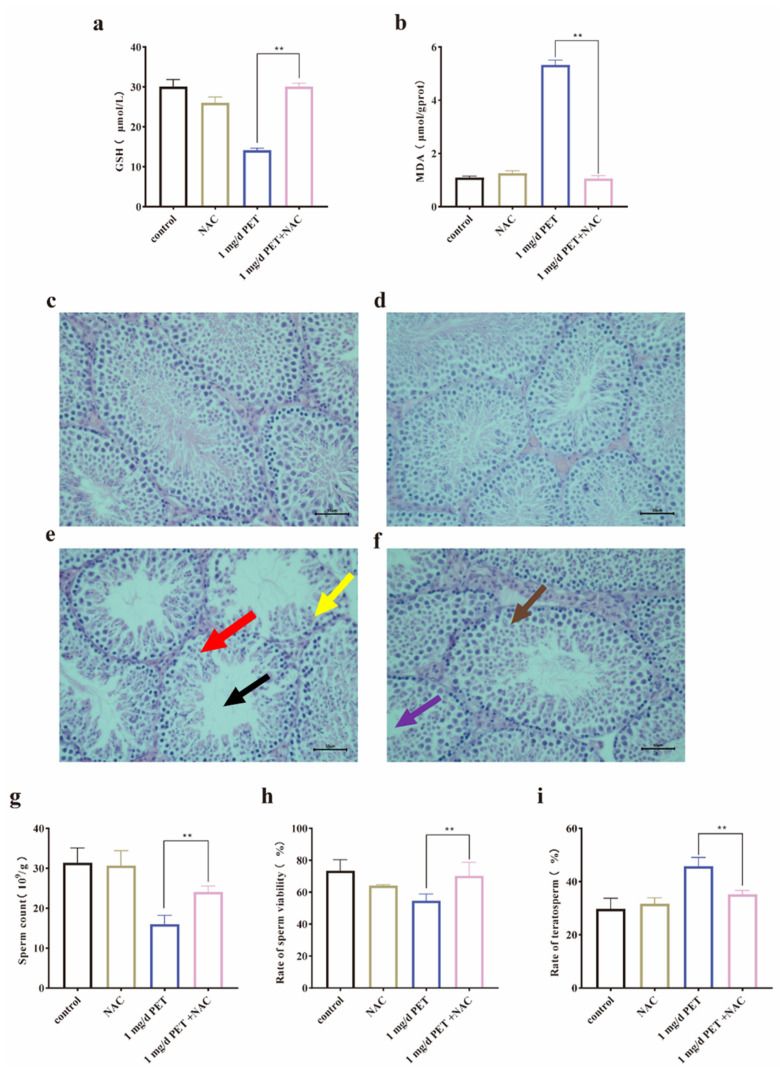
The pathological changes in the testicular tissue of mice after NAC antagonism. (**a**) GSH levels; (**b**) MDA levels; (**c**) control group; (**d**) NAC-treated group; (**e**) 1 mg/d PET MP group; (**f**) NAC intervention group; (**g**) sperm count levels; (**h**) sperm viability rate; and (**i**) teratosperm rate. The black arrow indicates that the spermatogenic tubule was empty. The red arrow indicates a disorder of the spermatogenic epithelium. The yellow arrow indicates a decrease in spermatogenic cells. The purple arrow indicates that the spermatogenic tubules are partially restored. The brown arrow indicates that the spermatogenic cells gradually recovered. Scale bar: 50 µm. *n* = 5 for all groups. Data are presented as the mean ± SEM. ** indicates *p* < 0.01 (compared with the control group).

To further verify the role of oxidative stress in PET MP exposure-induced reproductive damage in mice, NAC was specifically used to antagonize oxidative stress. As can be observed in Figure 7a,b, the GSH levels significantly increased (*p* < 0.01) and the MDA levels significantly decreased (*p* < 0.01) in the testicular tissues of the mice after NAC antagonism. Although there was still minor pathological damage compared with the control group (Figure 7c), the degree of this damage was greatly improved in the NAC intervention group (Figure 7f) compared with the PET MP-exposed group (Figure 7e). Moreover, NAC exposure did not cause any pathological damage (Figure 7b). Furthermore, it was also found that the antagonism of NAC significantly improved the sperm quality of the mice. As shown in Figure 7g,h, the sperm count and sperm survival were significantly up-regulated (*p* < 0.01), and, as shown in Figure 7i, the sperm malformation rate was down-regulated (*p* < 0.01). Therefore, it was concluded that oxidative stress played a key role in the PET MP exposure-induced reproductive injury of male mice.

### 3.4. The Role of the p38 Signaling Pathway in Male Reproductive Toxicity Induced by PET MPs

**Figure 8:**
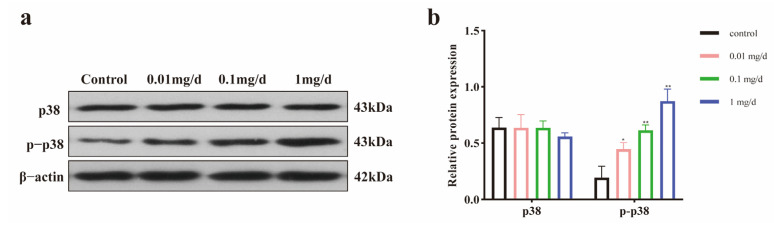
The activation of the p38 MAPK signaling pathway after PET MP exposure. (**a**) The expression of p38 and p-p38 in testis; (**b**) the quantification of p38 and p-p38 protein levels. β-actin was considered as the loading control. *n* = 5 for all groups. Data are presented as the mean ± SEM. * indicates *p* < 0.05 (compared with the control group). ** indicates *p* < 0.01 (compared with the control group).

The relative expression of p-p38, a key phosphorylated protein of the p38 MAPK signaling pathway, was found to be significantly increased (Figure 8a,b), indicating that PET MP exposure could activate the p38 signaling pathway.

**Figure 9:**
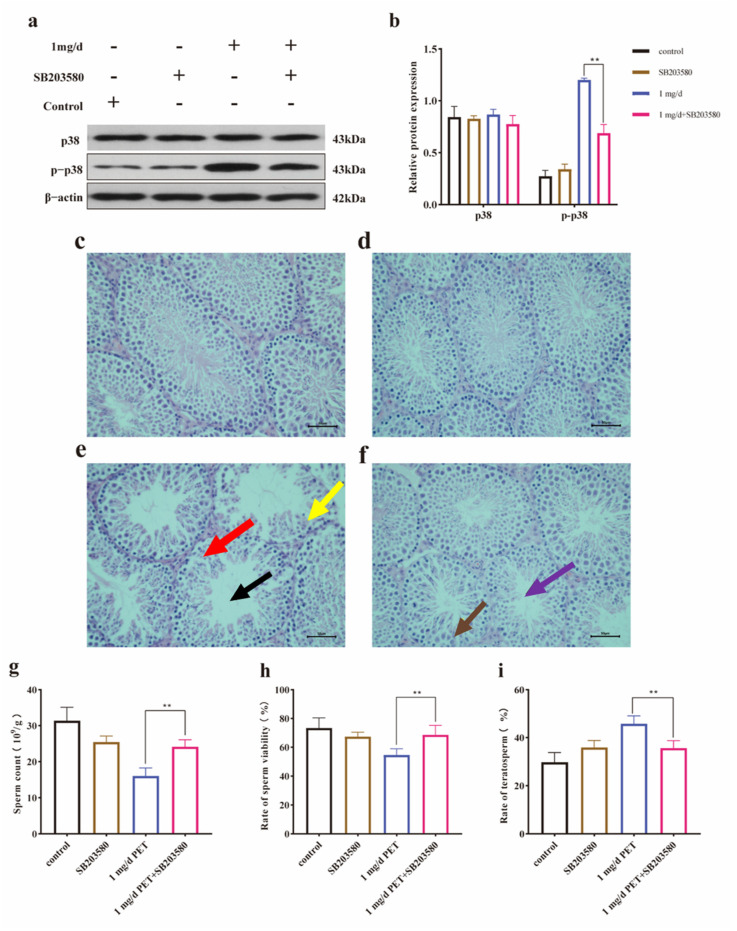
The pathological changes in testicular tissue after SB203580 blockade. (**a**) The expression of p38 and p-p38 in testis; (**b**) the quantification of p38 and p-p38 protein levels. β-actin was considered as the loading control. (**c**) Control group; (**d**) SB203580-treated group; (**e**) 1 mg/d PET MP group; (**f**) SB203580 intervention group; (**g**) the levels of sperm count; (**h**) the rate of sperm viability; and (**i**) the rate of teratosperm. The black arrow indicates that the spermatogenic tubule is empty. The red arrow indicates a disorder of the spermatogenic epithelium. The yellow arrow indicates a decrease in spermatogenic cells. The purple arrow indicates that the spermatogenic tubules are partially restored. The brown arrow indicates that the spermatogenic cells gradually recovered. Scale bar: 50 µm. *n* = 5 for all groups. Data are presented as the mean ± SEM. ** indicates *p* < 0.01 (compared with the corresponding intervention group).

To further verify the role of the p38 signaling pathway in the reproductive injury caused by PET MPs, SB203580 was used to specifically block the p38 signaling pathway. As shown in Figure 9a,b, the results of the Western blot assay revealed that the relative expression of phosphorylated protein p-p38 was effectively down-regulated by SB203580 (*p* < 0.01). As was observed in the histopathology presented in Figure 9c–f, the damage to the testicular tissue caused by PET MP exposure significantly improved after SB203580 blocking. As shown in Figure 9f, the spermatogenic tubules were partially restored and the spermatogenic cells gradually recovered compared with the PET MP-exposed group (Figure 9e). No significant pathological tissue injury was observed in the control group (1) or in the SB203580-treated group. Moreover, it was also found that sperm quality recovered significantly (*p* < 0.01) after specific blockade by SB203580. As shown in Figure 9g,h, sperm count and sperm survival were significantly up-regulated and, as shown in Figure 9i, the sperm malformation rate was down-regulated.

**Figure 10:**
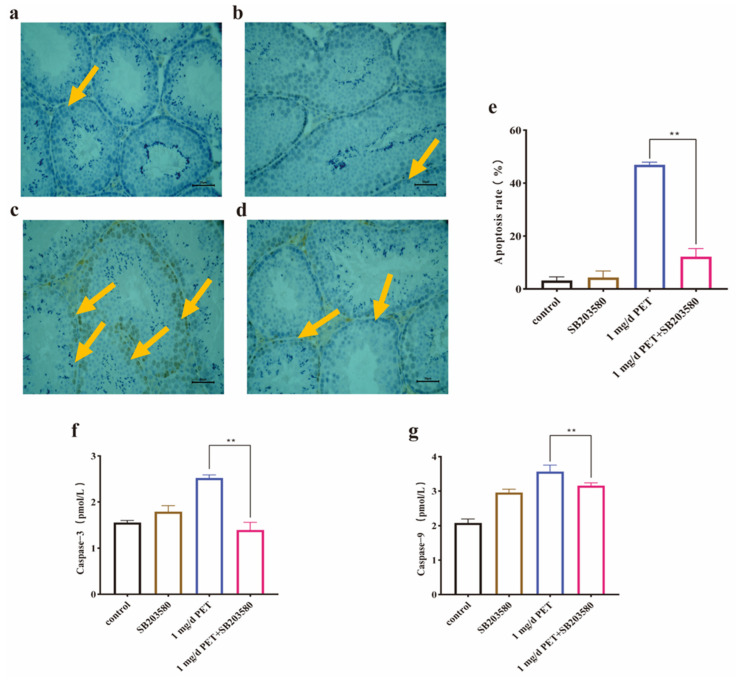
The effect of apoptosis and apoptosis factors after SB203580 blockade. (**a**) Control group; (**b**) SB203580-treated group; (**c**) 1 mg/d PET MP group; (**d**) SB203580 intervention group; (**e**) the quantitative analysis of apoptosis rate; (**f**) Caspase-3 levels; and (**g**) Caspase-9 levels. The orange arrow indicates the apoptotic cells. Scale bar: 50 µm. *n* = 5 for all groups. Data are presented as the mean ± SEM. ** indicates *p* < 0.01 (compared with the corresponding intervention group).

In addition, the results also showed that cell apoptosis was reversed. As shown in Figure 10a,b, cell apoptosis was very rare in the control and SB203580-treated groups. The brown area of apoptotic spermatogenic cells in the SB203580 intervention group (Figure 10d) was significantly less than that in the PET MP-exposed group (Figure 10c). Moreover, the apoptosis rate (Figure 10e) and the levels of the two apoptotic factors (Figure 10f,g) were significantly reduced (*p* < 0.01). Therefore, these findings demonstrated that the activation of the p38 signaling pathway could play an important role in the PET MP exposure-induced reproductive injury of male mice.

## 4. Discussion

The issue of environmental factors and reproductive impairment has been increasingly emphasized, and exposure to MPs might be one of the reasons for the general decline in male fertility over the past 50 years [40]. Some studies have found that exposure to different types of MPs can cause a variety of reproductive toxicity injuries, including male reproductive injury [38,41,42], female reproductive damage [43], and embryo toxicity [44]. PET, a type of polymer plastic, is used in 80% of plastic bottles and packaging materials [45] and has been demonstrated to be present in the human gastrointestinal tract [46], blood [14], and reproductive system [16]. However, the reproductive toxicity of PET MP exposure and its molecular mechanisms are currently unknown. The smaller the particle size of microplastics, the easier they break through the blood–testis barrier and enter the reproductive system, often causing greater reproductive toxicity [23,47]. Thus, in this study, PET MPs of 1 μm in particle size were chosen, with three doses of 0.01 mg/d, 0.1 mg/d, and 1 mg/d. Then, mice were exposed to these PET MPs for 42 days.

This study found that PET MPs could cause reproductive damage in male mice. Testicular histopathological damage and sperm quality have often been used to assess male reproductive injury. In this study, exposure to PET MPs was found to induce pathological changes in mice testicular tissues, such as the vacuolization of seminiferous tubules, a reduction in spermatogenic cells, and a disorganization of the seminiferous epithelium. Moreover, sperm quality was decreased by exposure to different doses of PET MPs, including a decrease in sperm count, a reduction in sperm survival, and an increase in the sperm malformation rate. Some studies have found that exposure to other types of MPs can also cause a variety of male reproductive toxicity injuries, including decreased sperm quality caused by PS MPs [41], associated reproductive hormone abnormalities induced by the bioavailability of testosterone decreasing caused by PA MPs [42], and testicular damage induced by PS MPs [38]. In addition, during sperm production, testosterone stimulation [48] and the protection of the blood–testosterone barrier [49] are essential. Relevant studies have shown that exposure to PS MPs can reduce testosterone levels in mice and destroy their blood–testosterone barrier, thus causing a decline in sperm quality [23]. Additionally, the development of spermatogenic cells at different stages affects the number of sperm, and poor development can lead to a decrease in sperm count [24]. Spermatogenic cell apoptosis can also be induced by PS MPs with a size of 5 μm through the p53 signaling pathway, thus causing reproductive toxicity in male mice [50]. It is inferred that spermatogenic cells of testicular tissue undergo apoptosis under the influence of PET MPs, which leads to a decrease in sperm count. Therefore, PET MPs cause reproductive damage in male mice through the combined result of various influencing factors. However, the underlying mechanism by which PET MPs affect sperm quality needs to be further clarified.

In order to resist oxidative damage, organisms activate their own antioxidant system. GSH can convert oxides into hydroxyl compounds, thus producing antioxidant effects [24]. In addition, MDA is a metabolite of oxygen radicals that can directly respond to the degree of lipid peroxidation in the organism [51]. Relevant research has indicated that oxidative stress might be one of the mechanisms involved in the decline in male reproductive function [52]. Our results showed that the GSH levels decreased and that the MDA levels increased in the testicular tissues after exposure to different doses of PET MPs, suggesting that the effects of oxidative damage were aggravated by exposure to PET MPs. To further investigate the role of oxidative stress in reproductive damage caused by PET MPs, antioxidant NAC, the precursor of GSH, was used in an intervention experiment. The results showed that NAC effectively alleviated the testicular and sperm damage caused by PET MPs. Previous studies have reported that PS MP exposure induced oxidative stress in testicular tissue, and the intervention of NAC was able to alleviate the decrease in GSH and the increase in MDA, which was consistent with the relevant results of this study [24]. Therefore, given the mechanistic role of oxidative damage in microplastic-induced reproductive toxicity, antioxidant preventive methods deserve public health attention.

The Mitogen-Activated Protein Kinase (MAPK) signaling pathway has also been shown to be closely associated with reproductive damage in terms of sperm quality [53], testicular function [54], levels of androgenic reproductive hormones [55], and the integrity of the blood–testis barrier [56]. Among them, the p38 signaling pathway is an important member of the MAPK family, which can act as a key mediator in the inflammatory signaling pathway [57] and mediate apoptosis by decreasing the activity of anti-apoptotic genes [39]. In this study, we found that the p38 signaling pathway was activated in the testicular tissues after exposure to PET MPs. In order to further clarify the effect of p38 activation in the reproductive damage and spermatogenic cell apoptosis induced by PET MPs, SB203580 was used for intervention experiments in this study. SB203580 is a cell-permeable inhibitor of p38; it has been reported to effectively inhibit the activation of the p38 signaling pathway caused by exposure to exogenous substances [34,35,58]. Our results showed that the activation of the p38 signaling pathway was inhibited after the SB203580 intervention, while testicular pathological damage, sperm quality, and cell apoptosis significantly improved. A relevant study found that the increased p-p38 expression level of male mice exposed to PS MPs could be significantly reduced by SB203580, which was consistent with the results in this study [24]. Thus, the p38 signaling pathway can play a key role in the reproductive toxicity induced by MPs, and it warrants further attention in future studies.

## 5. Conclusions

In summary, PET MP exposure was found to cause male reproductive impairment, mainly including sperm quality, pathological morphological changes in the testes, and spermatogenic cell apoptosis. More importantly, our study revealed that oxidative stress and the p38 signaling pathway could play important roles in male reproduction toxicity induced by PET MPs, thus providing reliable data for public protection strategies and further mechanism research. However, the following limitations of this study need to be further examined: (1) Due to the limitations of existing technology, MPs with small particle sizes (1 µm) cannot be detected. Therefore, PET MPs in testicular tissue were not detected in our study. (2) In this study, commercial PET MP standard products were used instead of naturally degraded PET MPs. In future studies, we plan to use naturally degraded PET MPs to fully investigate the combined toxicity of PET MPs and more closely simulate real environmental exposure.

## Data Availability

All data generated or analyzed during this study are included in this published article.
